# Noninvasive Computed Tomography-Based Quantification of Tumor Fibrosis Predicts Pancreatic Cancer Response to Gemcitabine/Nab-Paclitaxel

**DOI:** 10.34133/research.0937

**Published:** 2025-10-03

**Authors:** Qiuxia Yang, Yize Mao, Yulong Han, Kailai Li, Wanming Hu, Jianyao Zhou, Xuejun Gong, Shuxiang Huang, Rong Zhang, Lizhi Liu, Ningning Niu, Yixiong Li, Liandong Ji, Xiaoping Yi, Wufeng Xue, Dong Ni, Wenjun Mao, Peng Luo, Dong Luo, Jun Cheng

**Affiliations:** ^1^ Department of Radiology, State Key Laboratory of Oncology in South China, Guangdong Provincial Clinical Research Center for Cancer, Sun Yat-sen University Cancer Center, Guangzhou 510060, China.; ^2^ Department of Pancreatobiliary Surgery, State Key Laboratory of Oncology in South China, Guangdong Provincial Clinical Research Center for Cancer, Sun Yat-sen University Cancer Center, Guangzhou 510060, China.; ^3^ National-Regional Key Technology Engineering Laboratory for Medical Ultrasound, Guangdong Key Laboratory of Biomedical Measurements and Ultrasound Imaging, School of Biomedical Engineering, Thoracic Surgery Department of the First Affiliated Hospital, Shenzhen University Medical School, Shenzhen University, Shenzhen 518055, China.; ^4^ Medical UltraSound Image Computing (MUSIC) Lab, Shenzhen University, Shenzhen 518055, China.; ^5^ Marshall Laboratory of Biomedical Engineering, Shenzhen University, Shenzhen 518055, China.; ^6^ Department of Oncology, Zhujiang Hospital, Southern Medical University, Guangzhou 510282, China.; ^7^ Department of Pathology, State Key Laboratory of Oncology in South China, Guangdong Provincial Clinical Research Center for Cancer, Sun Yat-sen University Cancer Center, Guangzhou 510060, China.; ^8^ Department of Radiology, Affiliated Dongguan Hospital, Southern Medical University, Dongguan, China.; ^9^ Division of Pancreatic Surgery, Department of General Surgery, Xiangya Hospital, Central South University, Changsha, Hunan 410008, China.; ^10^ National Clinical Research Center for Geriatric Disorders, Xiangya Hospital, Central South University, Changsha, Hunan 410008, China.; ^11^ State Key Laboratory of Systems Medicine for Cancer, Ren Ji Hospital, Shanghai Cancer Institute, Shanghai Jiao Tong University School of Medicine, Shanghai, China.; ^12^ Department of Radiology, Xiangya Hospital, Central South University, Changsha, Hunan, China.; ^13^ School of Artificial Intelligence, Shenzhen University, Shenzhen 518060, China.; ^14^ National Engineering Laboratory for Big Data System Computing Technology, Shenzhen University, Shenzhen 518060, China.; ^15^ School of Biomedical Engineering and Informatics, Nanjing Medical University, Nanjing 211166, China.; ^16^ Department of Thoracic Surgery, the Affiliated Wuxi People’s Hospital of Nanjing Medical University, Wuxi People’s Hospital, Wuxi Medical Center, Nanjing Medical University, Wuxi 214023, China.; ^17^ Wuxi College of Clinical Medicine, Nanjing Medical University, Wuxi 214023, China.

## Abstract

Pancreatic ductal adenocarcinoma (PDAC) carries a dismal prognosis. Chemotherapy remains the mainstay for unresectable cases, yet regimens like AG (gemcitabine/nab-paclitaxel) exhibit heterogeneous efficacy. Tumor fibrosis has emerged as a potential predictor of treatment response but lacks validated noninvasive assessment methods. To address this, in this multicenter study, tumor fibrosis was quantified in 361 patients with resectable PDAC from SYSUCC, XYCSU, and TCGA cohorts using deep learning-based tissue segmentation on hematoxylin and eosin-stained whole-slide images. Fibrosis was defined as stromal proportion, and its association with overall survival (OS) was evaluated. Transcriptomic profiling was performed in 51 XYCSU cases to validate the biological relevance of fibrosis quantification. A radiomics model (RM) was then developed using preoperative contrast-enhanced computed tomography (CT) scans from SYSUCC to predict fibrosis and externally validated in XYCSU. Clinical utility was assessed in an independent cohort of 295 unresectable PDAC patients treated with AG, FOLFIRINOX, or SOXIRI. High fibrosis correlated with prolonged OS across resectable cohorts (all *P* < 0.05). Transcriptomic analysis revealed enrichment of fibrosis-related pathways in high-fibrosis tumors. The RM achieved an area under the curve of 0.718 (95% confidence interval: 0.627 to 0.823) in the external test set. Among patients receiving AG, those with CT-predicted high fibrosis had significantly longer progression-free survival (median: 6.23 versus 4.70 months, *P* = 0.037) and OS (13.37 versus 7.73 months, *P* = 0.002). No significant survival benefit was observed for high-fibrosis patients receiving FOLFIRINOX or SOXIRI. CT-based fibrosis quantification offers a robust, noninvasive biomarker for predicting AG efficacy in unresectable PDAC.

## Introduction

The incidence of pancreatic ductal adenocarcinoma (PDAC) continues to rise annually [1,2], with a 5-year survival rate of approximately 13% across all stages [1], despite advances in therapeutic strategies [3]. This poor prognosis is attributed not only to frequent late-stage diagnosis but also to the lack of effective treatments. Targeted therapies and immunotherapies have failed to improve overall survival (OS) in patients with unresectable PDAC [3,4], leaving chemotherapy as the primary systemic treatment option. Current first-line regimens, including FOLFIRINOX (a combination of 5-fluorouracil, leucovorin, irinotecan, and oxaliplatin) and AG (gemcitabine/nab-paclitaxel), achieve a median OS of approximately 12 months [5,6]. A recent network meta-analysis suggested FOLFIRINOX as the preferred regimen for patients with good performance status, while AG remains a viable alternative [5]. In clinical practice, the selection of systemic therapy must balance patient fitness with the potential efficacy of specific regimens.

Molecularly guided therapy selection is emerging as a promising strategy for PDAC. Recently, several biomarkers have been explored to assist in chemotherapy selection. Among these, certain biomarkers based on protein expression have been shown to predict sensitivity to the AG regimen. For instance, human equilibrative nucleoside transporter 1 (hENT1) has been extensively investigated as a predictor for gemcitabine efficacy. High hENT1 expression is associated with improved treatment response in both adjuvant [7] and advanced PDAC settings [8]. Beyond hENT1, low TUBB3 expression has also been identified as a favorable predictor of response to AG [9]. In addition, RNA-based biomarkers have also been developed to assess gemcitabine sensitivity. For example, high LAMC2 expression is associated with resistance to gemcitabine in unresectable PDAC [10]. Furthermore, the GemPred RNA signature has been shown to predict benefit from adjuvant gemcitabine in PDAC patients [11,12]. While these findings suggest that biomarkers could optimize patient selection for AG chemotherapy, current methods for assessing these proteins and RNA-based signatures face considerable limitations, including their reliance on biopsy specimens that are often compromised by PDAC’s dense stromal fibrosis, leading to sampling bias, which limited the clinical applicability. Thus, the need for noninvasive, reproducible imaging biomarkers to enable personalized prediction of chemotherapy response is urgent.

A hallmark histopathological feature of PDAC is its abundant desmoplastic stroma [13–15]. Emerging evidence suggests that AG may exhibit superior efficacy in tumors with high stromal content [16], highlighting the potential predictive value of stromal composition. However, current methods for assessing tumor fibrosis in advanced PDAC primarily rely on invasive biopsy-based immunohistochemical staining (e.g., collagen quantification), which suffers from sampling bias and fails to capture spatial heterogeneity.

**Fig. 1.**
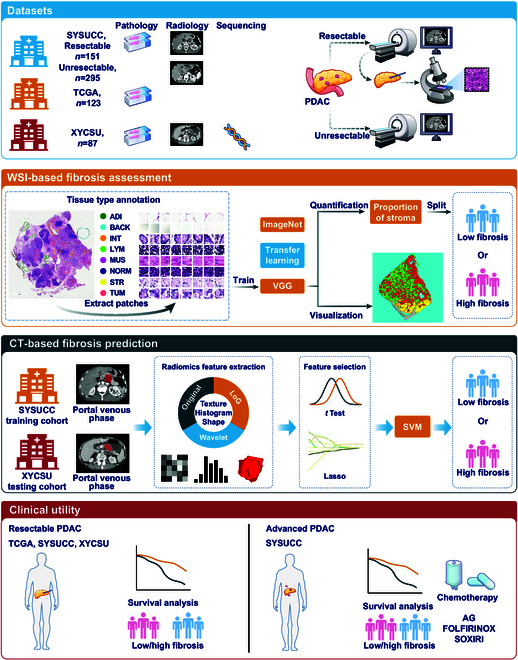
Overview of the study design.

To address these challenges, this study first established a deep learning-based fibrosis assessment method using whole-slide images (WSIs) in resectable PDAC cohorts, which served as the reference standard. The biological relevance of this method was further validated through RNA sequencing analysis. A radiomics model (RM) was then developed using preoperative contrast-enhanced computed tomography (CECT) scans from 2 centers to predict tumor fibrosis. Subsequently, in a large cohort of unresectable PDAC patients receiving chemotherapy, we evaluated the clinical utility of this CT-based fibrosis prediction model across multiple regimens (AG, FOLFIRINOX, and SOXIRI). This model enables noninvasive, whole-tumor quantification of tumor fibrosis, providing a novel tool for personalized therapeutic decision-making in PDAC. The overview of study design is shown in Fig. 1.

## Results

### Patient characteristics

**Fig. 2.**
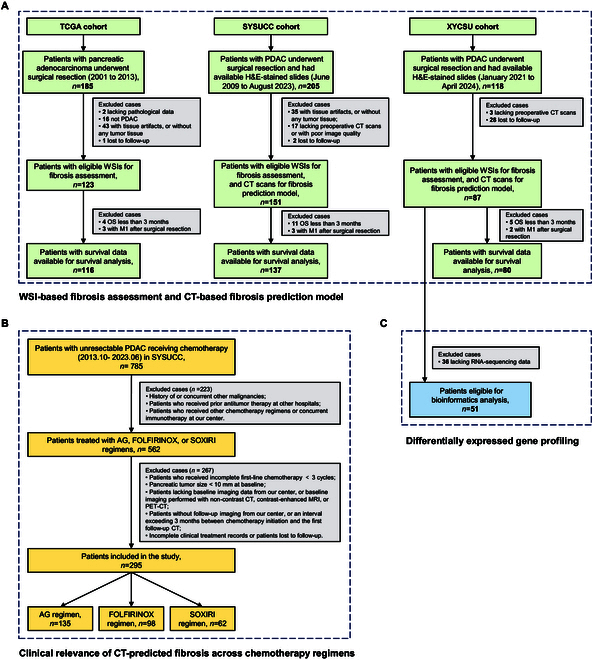
Flowchart of patient screening and enrollment for each study cohort. Different cohorts are collected for the following tasks: (A) WSI-based fibrosis assessment and CT-based fibrosis prediction model, (B) clinical relevance of CT-predicted fibrosis across chemotherapy regimens, and (C) differentially expressed gene profiling analysis. PDAC, pancreatic ductal adenocarcinoma; H&E, hematoxylin-eosin; WSI, whole-slide image; AG, gemcitabine/nab-paclitaxel; FOLFIRINOX, 5-fluorouracil, leucovorin, irinotecan, and oxaliplatin; SOXIRI, S-1, oxaliplatin, and irinotecan; M1, metastatic pancreatic ductal adenocarcinoma.

A total of 361 patients with resectable PDAC were included across 3 independent cohorts: 123 patients from the Cancer Genome Atlas cohort (TCGA, median age: 66 years; 56 women and 67 men), 151 from the Sun Yat-sen University Cancer Center cohort (SYSUCC, median age: 60 years; 62 women and 89 men), and 87 from the Xiangya Hospital of Central South University cohort (XYCSU, median age: 61 years; 40 women and 47 men) (Fig. 2A). Hematoxylin and eosin (H&E)-stained WSIs were available for all 3 cohorts, and RNA sequencing data were obtained for 51 patients in the XYCSU cohort (Fig. 2C). Preoperative CECT images were available for the SYSUCC and XYCSU cohorts. Detailed clinicopathological characteristics for the resectable cohorts are summarized in Table S1.

Additionally, 295 patients with unresectable PDAC (median age: 58 years; 118 women and 117 men) from SYSUCC received first-line chemotherapy, including 135 patients treated with AG, 98 with FOLFIRINOX, and 62 with SOXIRI (Fig. 2B). Patient characteristics are summarized in Table S2.

### Prognostic value of WSI-based fibrosis stratification across cohorts

**Fig. 3.**
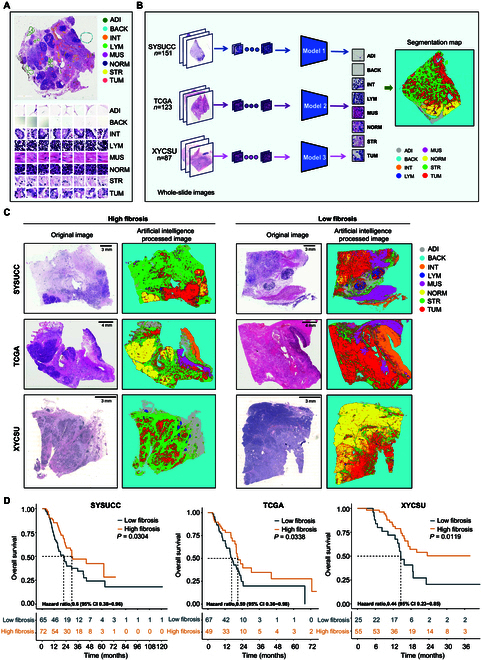
WSI-based fibrosis assessment. (A) Schematic illustration of manual annotations for 8 distinct tissue classes in whole-slide image. (B) Development of tissue classification models and visualization of segmentation results. (C) Representative segmentation maps from high- and low-fibrosis patients, with stroma shown in green. (D) WSI-based fibrosis stratification is significantly associated with overall survival across all cohorts.

The deep learning-based multiclass tissue classification models achieved high accuracies in all 3 surgical cohorts, with 8-class accuracies of 0.960 [95% confidence interval (CI): 0.949 to 0.969] in the TCGA cohort, 0.958 (95% CI: 0.947 to 0.967) in SYSUCC, and 0.971 (95% CI: 0.962 to 0.979) in XYCSU, evaluated on their respective test sets. Confusion matrix analysis revealed that the most frequent misclassifications occurred between muscle and stromal tissues, and between adipose and background regions, likely due to morphological similarity (Fig. S1). Representative WSIs illustrating high- versus low-fibrosis phenotypes in each cohort are shown in Fig. 3C. The tissue decomposition results were highly concordant with expert histopathological interpretation, confirming the model’s strong capability to identify stromal components in WSIs.

Across all the 3 cohorts, the patients in the high-fibrosis group (stromal proportion > 43%) consistently exhibited significantly superior OS (Fig. 3D). In TCGA, the median OS was 20.90 months in the high-fibrosis group (*n* = 49) versus 16.60 months in the low-fibrosis group (*n* = 67) [hazard ratio (HR) = 0.59 [95% CI, 0.36 to 0.98], *P* = 0.034]. In SYSUCC, high-fibrosis patients (*n* = 72) had a median OS of 29.8 months compared to 21.53 months in the low-fibrosis group (*n* = 65) (HR = 0.60 [0.38 to 0.96], *P* = 0.030). In XYCSU, the low-fibrosis group (*n* = 25) had a median OS of 14.53 months, while the median OS was not reached in the high-fibrosis group (*n* = 55), which achieved a 3-year survival rate of 50.38% (HR = 0.44 [0.23 to 0.85], *P* = 0.012).

### Fibrosis groups show distinct pathway and transcriptomic profiles

**Fig. 4.**
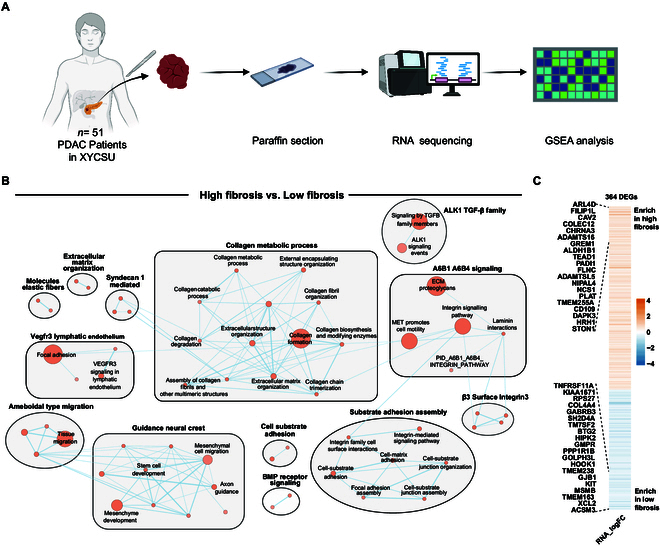
Fibrosis-associated differentially expressed gene profiling. (A) Analytical workflow. Fifty-one PDAC patients from XYCSU with transcriptomic data were stratified into high- and low-fibrosis groups based on WSI-derived fibrosis assessment. (B) Enrichment network visualization. Nodes represent significantly enriched gene sets, with node size proportional to gene count. Edges indicate overlap between gene sets, with thickness scaled to shared gene count. (C) Volcano plot of 364 fibrosis-associated DEGs (red: up-regulated in high-fibrosis; blue: down-regulated).

Gene set enrichment analysis (GSEA), visualized using EnrichmentMap, revealed significantly enriched biological processes in high-fibrosis tumors (Fig. 4B and Table S3). Among these, the collagen metabolic process emerged as the most prominent functional module, encompassing collagen biosynthesis, degradation, trimer assembly, and extracellular matrix (ECM) organization. These findings not only corroborate the up-regulation of multiple ECM-related differentially expressed genes (DEGs) but also underscore ECM remodeling as a central hallmark of tumor-associated fibrosis. Additionally, the integrin-mediated cell adhesion pathway was markedly activated, highlighting the importance of cell–matrix interactions in fibrogenesis. Key features included focal adhesion formation, matrix adhesion assembly, and coordinated regulation of A6B1/A6B4 signaling pathways. Furthermore, the independently clustered ALK1 transforming growth factor-β (TGF-β) family signaling module reinforced this pathway’s central regulatory role in fibrogenesis.

Transcriptomic analysis identified 364 significant DEGs between high- and low-fibrosis groups (Fig. 4C and Table S4), with 220 genes up-regulated and 144 genes down-regulated in the high-fibrosis group compared to the low-fibrosis group. The most significantly up-regulated genes were ARL4D, FILIP1L, CAV2, COLEC12, and CHRNA3. Notably, pronounced up-regulation of ECM-remodeling genes (ADAMTS16, ADAMTS5, and COLEC12) showed high concordance with characteristic fibrotic histopathology. Additionally, GREM1 (a bone morphogenetic protein antagonist), ALDH1B1 (a member of aldehyde dehydrogenase family), and FLNC (an actin cytoskeleton tissue protein) genes with tissue repair and fibrosis regulation functions also showed significantly high expression. Conversely, several genes including TNFRSF11A (RANK receptor), KIAA1671, RPS27, and COL4A4 were significantly down-regulated in the high-fibrosis group.

Compared to the low-fibrosis group, expression of COL1A1 (*P* = 0.042) and COL1A2 (*P* = 0.032) was significantly higher in the high-fibrosis group, whereas COL4A4 expression (*P* = 0.041) was lower (Fig. S2A and B). Immune infiltration analysis further revealed no significant differences in most cell subsets between the fibrosis groups, except for fibroblasts (*P* = 0.008) (Fig. S2C).

Correlation analysis indicated no significant association between stromal proportion and the expression of hENT1 (SLC29A1), TUBB3, or LAMC2 (all *P* > 0.05; Fig. S3A). Similarly, when comparing the high- and low-fibrosis groups, no significant differences were observed in the expression levels of these genes (all *P* > 0.05; Fig. S3B).

### CT-based RM predicts tumor fibrosis with prognostic relevance in resectable PDAC

**Fig. 5.**
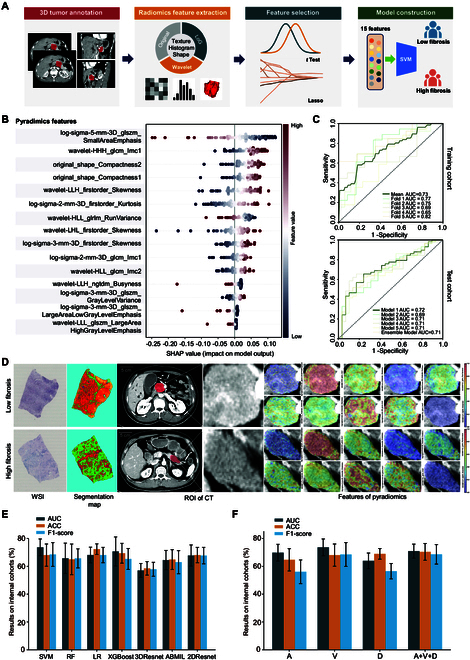
CT-based fibrosis prediction. (A) Workflow of radiomics analysis. (B) SHAP summary plot showing the top 15 radiomic features contributing to fibrosis prediction. (C) Fivefold cross-validation performance of the CT-based fibrosis prediction model on the training (SYSUCC) and test (XHCSU) cohorts. (D) Representative contrast-enhanced CT (CECT) images with discriminative textural feature maps in high- and low-fibrosis patients. (E) Performance comparison of different fibrosis prediction models using features extracted from venous-phase CT images. (F) Performance comparison of different CT imaging phases. SVM classifiers were built using features extracted from arterial-, venous-, and delayed-phase images, as well as their average combination. AUC, area under the receiver operating characteristic curve; ACC, accuracy; A, arterial phase image; V, venous phase image; D, delayed phase image.

Fifteen fibrosis-associated radiomic features were identified (Fig. 5B and Table S5) to construct a predictive RM. SHapley Additive exPlanations (SHAP) analysis revealed that log-sigma-5-mm-3D_glszm_SmallAreaEmphasis contributed most substantially to predictions (Fig. 5B). Notably, lower values of this feature (blue distribution) were strongly associated with a higher probability of fibrosis. The second most contributive feature, wavelet-HHH_glcm_Imc1, showed a positive correlation between higher values (red distribution) and fibrosis, indicating complex spatial heterogeneity of gray-level intensities. Additionally, high values of log-sigma-3-mm-3D_glszm_LargeAreaHighGrayLevelEmphasis suggested the presence of extensive fibrotic regions. Collectively, these textural features captured key aspects of intratumoral textural heterogeneity and multiscale morphological patterns associated with fibrotic burden. Furthermore, SHAP analysis highlighted the role of the morphological feature original_shape_Compactness, implicating tumor shape regularity and compactness as critical contributors to fibrosis prediction. Representative CT images comparing high- and low-fibrosis patients, along with visualizations of discriminative textural features, are presented in Fig. 5D. These results validate the model’s radiobiological plausibility and interpretability from an imaging biomarker perspective.

In 5-fold cross-validation within the SYSUCC surgical cohort, the model achieved a mean area under the curve (AUC) of 0.736 ± 0.06, with the best-performing fold 1 model reaching an AUC of 0.773 (Fig. 5C and Table S6). External validation in the independent XYCSU cohort demonstrated stable predictive performance across the 5 cross-validated models, with AUC values ranging from 0.692 to 0.718 (SD: 0.0103), indicating strong model robustness. An ensemble approach via mean probability aggregation achieved an integrated AUC of 0.709 (95% CI: 0.619 to 0.819). Notably, the fold 1 model also yielded the highest external validation performance, with an AUC of 0.718 (95% CI: 0.627 to 0.823).

CT-predicted fibrosis stratification showed distinct survival in both the SYSUCC and XYCSU surgical cohorts (Fig. S4). In SYSUCC, high-fibrosis patients (*n* = 80) showed a longer median OS of 29.77 months compared to 22.27 months in the low-fibrosis group (*n* = 57), although the difference did not reach statistical significance (*P* = 0.056). The XYCSU cohort demonstrated a statistically significant survival difference (HR = 0.44 [95% CI, 0.23 to 0.85], *P* = 0.013), with high-fibrosis patients (*n* = 43) achieving a median OS of 23.43 months versus 14.50 months in the low-fibrosis group (*n* = 37).

Comparative analyses showed that deep learning-based models exhibited overfitting and poorer generalizability compared with the RMs (Fig. 5E and Table S7). Additionally, venous-phase CT images provided the highest predictive performance among individual imaging phases (Fig. 5F and Table S7).

### Clinical utility of CT-based fibrosis prediction across chemotherapy regimens

**Fig. 6.**
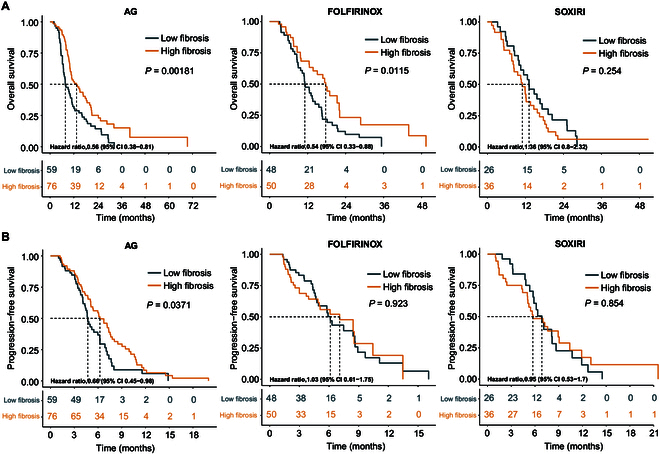
Clinical utility of CT-based fibrosis prediction across chemotherapy regimens. (A) Overall survival stratified by CT-predicted fibrosis status (high versus low) in patients receiving AG, FOLFIRINOX, or SOXIRI. (B) Progression-free survival stratified by CT-predicted fibrosis status (high versus low) under the same treatment regimens.

In the unresectable SYSUCC cohort, CT-predicted fibrotic status was associated with differential chemotherapy outcomes across AG, FOLFIRINOX, and SOXIRI regimens (Fig. 6).

Patients receiving AG demonstrated significantly better outcomes in the high-fibrosis group compared with the low-fibrosis group. Median OS was notably prolonged (13.37 months [95% CI, 10.2 to 18.67] versus 7.73 months [6.77 to 11.17], HR = 0.56 [95% CI, 0.38 to 0.81], *P* = 0.002), along with improved progression-free survival (PFS; median PFS, 6.23 months [5.10 to 7.60] versus 4.70 months [4.30 to 6.23], HR = 0.66 [0.45 to 0.98], *P* = 0.037).

In the FOLFIRINOX group, high-fibrosis patients also demonstrated marked OS improvement (median OS, 17.70 months [13.73 to 22.10] versus 11.10 months [9.77 to 15.53], HR = 0.54 [0.33 to 0.88], *P* = 0.012). However, PFS was not significantly different between the 2 groups (median PFS, 7.07 versus 6.13 months, *P* = 0.923).

Conversely, among patients treated with SOXIRI, no significant survival benefit was observed with respect to fibrotic status. Median OS was 11.03 months for high-fibrosis versus 13.17 months for low-fibrosis patients (*P* = 0.254), while median PFS was 5.70 versus 6.80 months (*P* = 0.854).

**Fig. 7.**
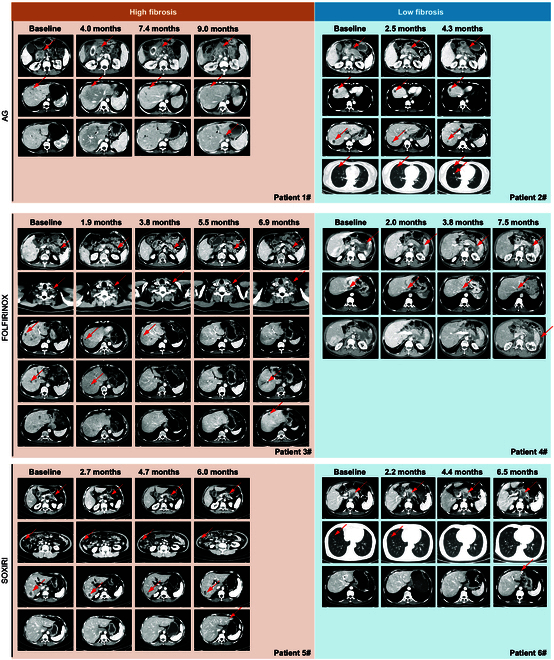
Serial CECT assessment of treatment response stratified by fibrosis status (high versus low) and chemotherapy regimens (AG, FOLFIRINOX, and SOXIRI). The detailed legends of each image were described in Supplementary Methods.

Figure 7 illustrates representative cases showing tumor response following chemotherapy in 6 patients stratified by fibrotic status (high versus low) and treated with AG, FOLFIRINOX, or SOXIRI. These visual comparisons further support the heterogeneity in treatment outcomes based on fibrosis level.

## Discussion

Quantitative assessment of tumor fibrosis holds substantial potential for improving patient stratification and optimizing treatment decision-making PDAC [16–18]. This multicenter study demonstrated that WSI-based fibrosis quantification was significantly associated with OS in resectable PDAC, with higher fibrosis correlating with improved outcomes. Furthermore, transcriptomic analysis comparing high- and low-fibrosis groups revealed enrichment of fibrotic-related pathways, supporting the biological relevance of this stratification. The RM constructed using CECT enabled accurate noninvasive quantification of tumor fibrosis and demonstrated robust predictive performance in an independent external test cohort. Notably, CT-predicted tumor fibrosis was significantly associated with chemotherapy response in unresectable PDAC. Among patients receiving the AG regimen, the high-fibrosis group exhibited significantly better PFS and OS. In contrast, no significant difference in PFS was observed between high- and low-fibrosis groups among patients treated with FOLFIRINOX or SOXIRI. These findings underscore the prognostic and predictive value of CT-based fibrosis estimation, particularly in identifying patients most likely to benefit from AG therapy.

Pancreatic stellate cells, activated by tumor cells, drive peritumoral fibrosis and the formation of a dense desmoplastic stroma [13,14]. This fibrotic microenvironment acts as a physical barrier, impeding angiogenesis and limiting chemotherapeutic drug delivery [13,19–21]. Nab-paclitaxel, a key component of the AG regimen, has been shown to reduce cancer-associated fibroblasts and tumor fibrosis [22–24], enhancing gemcitabine delivery by increasing stromal permeability [25]. Preclinical studies confirmed that stroma depletion promoted drug delivery to cancer cells [26], and the metastatic pancreatic adenocarcinoma clinical trial (MPACT) further validated significant tumor regression benefits with the AG regimen in metastatic PDAC [27]. These findings suggest that therapies targeting the tumor stroma may be particularly effective in tumors with high fibrosis.

However, traditional fibrosis assessment relies on Masson’s trichrome staining or immunohistochemistry of surgical specimens, which is often impractical in advanced cases due to limited biopsy tissue and substantial stromal heterogeneity. Shi et al. [16] reported that the strain ratio (SR), measured by endoscopic ultrasound elastography (EUS-EG), was positively associated with the stromal proportion in resected pancreatic cancer specimens. Moreover, patients with high SR exhibited a better response to the AG regimen. Despite this potential, EUS-EG is not routinely used to monitor treatment response in PDAC patients undergoing chemotherapy due to procedural complexity and patient intolerance to endoscopy. These limitations underscore the clinical need for noninvasive imaging approaches to quantify tumor fibrosis [28], particularly for guiding treatment strategies in patients with advanced PDAC.

CECT is the guideline-recommended modality for assessing resectability and staging in PDAC [29]. PDAC is typically characterized by low microvascular density and a progressive enhancement pattern, with the venous phase providing maximal tumor contrast for lesion detection [30]. Furthermore, most radiomic features are highly sensitive to variations in image intensity [31,32], supporting the feasibility of using venous-phase CT images to construct an RM for fibrosis prediction. This study addressed the limitations of subjective visual assessment by employing a deep learning algorithm to automatically classify 8 tissue types on WSIs. Our model demonstrated excellent multi-class tissue identification accuracy (>95%) across 3 cohorts. Computational pathology analysis enhanced reproducibility and enabled objective fibrosis quantification [33]. Using the surgical WSI-derived fibrosis stratification as the reference standard, we developed an RM based on preoperative venous-phase CT images to predict tumor fibrosis in resectable PDAC. This model demonstrated robust performance in an external test cohort, confirming its potential for noninvasive and accurate tumor fibrosis quantification.

Several prior studies have explored similar approaches. Meng et al. [34,35] developed CT- or magnetic resonance (MR)-based RMs to predict the tumor–stroma ratio in pancreatic cancer. However, both studies were conducted at single center. Shi et al. [17] also constructed a preoperative CT-based RM predicting tumor fibrosis (AUC = 0.760 in the training set and 0.656 in the test set) but did not further validate its clinical utility. Distinct from these previous studies, our work not only pioneered the artificial intelligence (AI)-automated quantification of the pathological metric “stromal proportion” in PDAC and validated its prognostic relevance through multicenter analysis but also developed a routine CT-based RM for its noninvasive assessment. Crucially, we validated this model’s predictive value for the treatment efficacy of the AG regimen in a chemotherapy cohort, providing direct clinical evidence to guide precision AG therapy selection. Importantly, transcriptomic analysis revealed significant enrichment of key molecules and interacting pathways involved in fibrosis progression in the high-fibrosis group. These findings not only validate the reliability of our fibrosis stratification but also identify potential stroma-targeted therapeutic strategies.

The prognostic value of tumor fibrosis in advanced PDAC varies across different chemotherapy regimens. Patients with high fibrosis receiving the AG regimen exhibited significantly longer PFS compared to those in the low-fibrosis group, whereas no such association was observed in patients treated with FOLFIRINOX or SOXIRI. Specifically, AG-treated patients with high fibrosis achieved a median PFS of 6.23 months, which was comparable to that of FOLFIRINOX-treated patients (7.07 months in the high-fibrosis group). These findings corroborate the work of Shi et al. [16], who reported a similar survival advantage for locally advanced PDAC patients with stroma-rich tumors treated with AG. The observed differential response underscores the critical importance of fibrosis-guided treatment selection, particularly given AG’s limited efficacy in stroma-poor tumors [16]. Based on these results, AG may be considered a preferred first-line option for high-fibrosis patients, offering efficacy comparable to FOLFIRINOX but with a more favorable toxicity profile. Prospective clinical trials are warranted to validate this fibrosis-guided therapeutic stratification strategy.

In resectable PDAC, our study adds to growing evidence supporting the tumor-suppressive role of stromal fibrosis. High-fibrosis patients consistently demonstrated significantly improved OS across 3 WSI-based cohorts, in line with multiple previous studies [18,28,36–40]. Although stroma has traditionally been regarded as a tumor-promoting component [15,41], accumulating evidence suggests that it also represents a reactive process to carcinogenesis, with dual regulatory effects—both promoting and restraining tumor progression [14,15,42–45]. For example, while collagen I could suppress PDAC growth through mechanical and immune modulation [46], tumor-derived collagen IV enhances proliferation and hinders drug delivery [47]. Our results showed that, compared to the low-fibrosis group, expression of COL1A1 and COL1A2 was significantly elevated in the high-fibrosis group, while COL4A4 expression was lower. This expression pattern partially explains why the high-fibrosis group was associated with better outcomes in resectable PDAC. Moreover, other studies have shown that stromal depletion—through Hedgehog inhibition, epithelial Sonic hedgehog deletion, fibroblast ablation, or lysyl oxidase-like 2 inhibition—can increase tumor vascularity, accelerate metastasis, and shorten survival [48–52]. Together, our findings reinforce the tumor-suppressive role of stroma in resectable PDAC and establish tumor fibrosis grading as a robust prognostic biomarker.

This study has several limitations. First, the retrospective design introduces potential selection bias. Some surgical patients did not undergo preoperative CECT at our institution [having received contrast-enhanced MR imaging (MRI) or external imaging], which may contribute to selection bias. Second, the modest sample sizes in the surgical cohort and chemotherapy subgroups necessitate validation through multicenter, large-scale prospective studies. Third, our fibrosis quantification was based on a single WSI per patient, which may not fully capture the impact of intratumoral heterogeneity. Based on these retrospective results, future prospective clinical trials should validate the predictive value of CT-derived tumor fibrosis for the AG regimen and assess its utility in guiding first-line therapy selection for patients with locally advanced and metastatic disease. Additionally, identifying imaging biomarkers predictive of response to other regimens such as FOLFIRINOX remains a critical research priority.

In conclusion, this study presents a CT-based, noninvasive tool for estimating tumor fibrosis, using WSI-derived fibrosis quantification as the reference standard in resectable PDAC cohorts. More importantly, CT-predicted tumor fibrosis was further validated as a promising predictive biomarker for treatment efficacy of the AG regimen in unresectable cases. These findings provide a foundation for individualized chemotherapy decision-making in PDAC, with the potential to improve patient outcomes while reducing unnecessary toxicity in nonresponders.

## Methods

### Patients and study design

Figure 1 presents an overview of the entire research process. This multicenter study utilized multimodal data from patients across 2 tertiary academic medical centers and a publicly available dataset. The study protocol received approval from the institutional review boards at all participating centers, with the requirement for patient informed consent waived.

Figure 2 illustrates the patient recruitment flowchart. Patients with PDAC who underwent surgical resection were collected from 3 centers: SYSUCC (June 2009 and August 2023), XYCSU (January 2021 to April 2024), and TCGA database (https://portal.gdc.cancer.gov/) (2001 to 2013) (Fig. 2A). Inclusion criteria were as follows: (a) pathologically confirmed PDAC by resection; (b) no prior chemotherapy or radiotherapy; (c) availability of at least one H&E-stained slide; (d) for SYSUCC and XYCSU cohorts, preoperative CECT performed within 2 weeks before surgery; and (e) complete clinical data and survival follow-up records. Detailed exclusion criteria for each cohort are provided in Supplementary Methods. In total, the TCGA cohort included 123 patients who underwent surgical resection. The SYSUCC and XYCSU cohorts included 151 and 87 patients, respectively, who underwent both CECT and surgical resection.

The XYCSU cohort included a genomic subset of 51 patients with clinicopathological data, preoperative CECT scans, and RNA sequencing data (Fig. 2C). This genomic dataset was utilized for GSEA [53] to investigate biological pathways associated with tumor fibrosis.

Additionally, patients with unresectable PDAC receiving first-line chemotherapy at SYSUCC between October 2013 and June 2023 were included (Fig. 2B), with inclusion criteria comprising: (a) pathologically confirmed PDAC, locally advanced or metastatic; (b) receiving chemotherapy of AG, FOLFIRINOX, or SOXIRI regimens based on patient performance status [5,29,54–57] for at least 3 cycles; (c) baseline CECT and ≥1 post-treatment imaging evaluation for efficacy assessment; and (d) complete clinical data and survival follow-up records. The SYSUCC chemotherapy cohort consisted of 295 unresectable patients who underwent both baseline CECT and chemotherapy. Detailed descriptions of patient treatment regimens are provided in Supplementary Methods.

As shown in Fig. 1, the study initially quantified tumor fibrosis using histopathological images from 3 surgical cohorts (TCGA, SYSUCC, and XYCSU). Survival analysis identified the optimal prognostic cutoff value to stratify patients into high- and low-fibrosis groups. Based on these pathological stratification labels, a preoperative CT-based fibrosis prediction model was developed in the SYSUCC cohort and externally validated in the XYCSU cohort. Finally, this CT-based fibrosis prediction model was applied to the SYSUCC chemotherapy cohort to noninvasively quantify tumor fibrosis and validate its correlations with chemotherapy response and survival outcomes across chemotherapy regimens.

### WSI-based fibrosis assessment

#### Dataset annotation and preparation

Given the histological complexity of PDAC, models were trained to distinguish 8 tissue classes in WSIs: tumor tissue, cancer-associated stroma, lymphocytes, normal pancreatic tissue, adipose tissue, small intestinal acinar tissue, muscle, and background (Fig. 3A). WSI preprocessing details are provided in Supplementary Methods.

Expert annotation was performed on a subset of WSIs to facilitate supervised learning. Specifically, 19 WSIs from TCGA, 20 from SYSUCC, and 8 from XYCSY were selected for manual annotation. A board-certified pancreatic pathologist (pathologist 1, with >8 years of experience in pancreatic pathology) delineated tumor core regions in Sedeen Viewer (v5.4.4) and labeled 8-class tissues using Aperio ImageScope (v12.4.6). Annotations were independently reviewed by another experienced pathologist (pathologist 2, also with >8 years of pancreatic pathology experience), with discrepancies resolved through consensus. Tumor cores were defined as regions with maximal tumor cellularity, allowing minimal admixture of normal, lymphoid, and muscular tissues while excluding necrotic or inflammatory areas.

Nonoverlapping patches of 224 × 224 pixels were extracted from annotated WSIs using a sliding window approach, which served as input for training and validating the tissue recognition model. For each cohort, 3 WSIs were randomly selected for testing, with 200 patches per tissue class (1,600 total) extracted to form the test set. The remaining WSIs constituted the training sets, from which 245,752 patches were generated from 16 WSIs in the TCGA cohort, 258,041 patches from 17 WSIs in SYSUCC, and 18,402 patches from 5 WSIs in XYCSU.

#### Development of tissue recognition model

A deep learning model based on a VGG19 architecture was employed to perform tissue classification. The model was initialized with weights pretrained on the ImageNet database (www.image-net.org), and its final fully connected layer was replaced with an 8-dimensional (8D) output to accommodate our tissue classification task. Model fine-tuning was performed using stochastic gradient descent with momentum (SGDM), with random horizontal/vertical flipping for data augmentation. To address staining variations across TCGA, SYSUCC, and XYCSU cohorts, independent tissue recognition models were developed for each cohort (Fig. 3B). The TCGA and SYSUCC models were trained independently using identical architectures and hyperparameters, while the XYCSU model was fine-tuned from the TCGA model.

During tissue quantification, the model outputs an activation matrix from the SoftMax layer for each image patch, representing the class probabilities (range, 0 to 1) across the 8 tissue types. For each WSI, the patch-level outputs were aggregated into a consolidated matrix of size [H/72, W/72, 8], where H and W represent the height and width of the original WSI, and 8 denotes the number of tissue classes.

To visualize tissue segmentation results, distinct RGB values were assigned to each tissue class. Final color maps were generated using activation-weighted summation of class-specific RGB values across image patches (Fig. 3B). The color mapping is defined by the following formula:

$$\mathrm{Seg}\_\mathrm{map}=\sum_{i=1}^{c}A\left[H,\;W,\;i\right]*\text{Colors}\left[i,\;1,\;1,\;3\right]$$ (1)

where *A* represents the activation matrix, *c* = 8 corresponds to the number of tissue classes, and *Colors* denotes predefined RGB values for each class.

Models were developed using MATLAB 2017b’s Deep Learning Toolbox, with distributed training across 4 NVIDIA GeForce RTX 1080Ti GPUs. Key training parameters included a mini-batch size of 360, learning rate of 3 × 10^−4^, 8 training epochs, SGDM optimizer (momentum = 0.9), and L2 regularization (λ = 1 × 10^−4^) to ensure stable convergence while balancing training efficiency and generalization performance.

#### Fibrosis quantification and grading threshold determination

To enable precise fibrosis quantification from WSIs, we applied a sliding window approach to generate overlapping 224 × 224-pixel patches from tumor core regions in all WSIs across the TCGA, SYSUCC, and XYCSU cohorts. These patches were processed using cohort-specific tissue recognition models to classify each image tile into one of 8 predefined tissue types. SP, representing the extent of tumor fibrosis, was then calculated for each patient as the ratio of patches classified as stromal tissue to the total number of patches within the annotated tumor core.

The median values of stromal proportion were 43% (range: 3% to 84%) in SYSUCC, 39% (range: 8% to 77%) in TCGA, and 49% (range: 11% to 86%) in XYCSU. To determine the fibrosis threshold for stratifying patients into high- and low-fibrosis groups, we systematically evaluated 61 candidate thresholds ranging from the 20th to the 80th percentile with a step size of 1%. More than half of these thresholds achieved statistically significant prognostic separation (61% for SYSUCC, 59% for TCGA, and 74% for XYCSU), highlighting the robust prognostic value of WSI-based fibrosis quantification. We ultimately selected 43%, the median value of stromal proportion of the largest SYSUCC cohort, as a universal threshold. This choice maximized statistical power by ensuring balanced group sizes for downstream analyses. Moreover, this threshold consistently yielded significant survival stratification in all 3 cohorts (*P* < 0.05), demonstrating its cross-cohort generalizability. Using a single, universal threshold also facilitates clinical translation by avoiding the need for center-specific calibration.

#### Association between tumor fibrosis and survival in resectable PDAC

To minimize perioperative mortality bias, only patients with OS ≥ 3 months were included in the survival analysis. OS differences between the high- and low-fibrosis groups were compared across the 3 surgical cohorts: TCGA, SYSUCC, and XYCSU. The study endpoint for surgical cohorts was OS, defined as the interval from the date of surgery to the date of death from any cause or the date of the last follow-up. For the in-house cohorts (SYSUCC and XYCSU), survival data were retrieved from electronic medical records. Kaplan–Meier curves and log-rank tests were used to evaluate survival differences between fibrosis groups.

### Transcriptomic profiling of fibrosis-associated DEGs

In the XYCSU cohort, RNA sequencing data were available for 51 patients, enabling downstream transcriptomic analysis (Fig. 4A).

#### Differential gene expression analysis

Transcriptomic differential expression analysis was performed using DESeq2 (v1.30.1) with significance thresholds of |log₂FC| >1 and adjusted *P* value <0.05. This approach identified 364 DEGs, encompassing both up-regulated and down-regulated genes. Expression patterns were visualized through a heatmap generated with pheatmap (v1.0.12), which annotated the top 20 most significantly up-regulated and down-regulated DEGs. Expression values were *z*-score normalized and depicted using an orange-to-blue color gradient (warm-to-cool scale).

#### Gene set enrichment analysis

GSEA (v4.1.0) [53] was applied to elucidate functional implications of transcriptional alterations in high- versus low-fibrosis groups. Unlike conventional enrichment methods, GSEA circumvents predefined differential expression thresholds by analyzing genome-wide expression trends, enabling detection of subtle yet biologically significant patterns. Curated gene sets from Gene Ontology (GO: Biological Process, Molecular Function, Cellular Component), Kyoto Encyclopedia of Genes and Genomes (KEGG), and Reactome databases were utilized. Genes were ranked by log₂FC, and enrichment scores of each gene set were computed using a weighted enrichment statistic. Statistical significance was assessed via 1,000 permutations with Benjamini–Hochberg false discovery rate (FDR) correction (significant threshold: *q* < 0.01).

#### Enrichment network visualization

To delineate functional associations among enriched pathways, an enrichment network was constructed using Cytoscape (v3.8.2) with the EnrichmentMap plugin. GSEA results (FDR *q* < 0.01) were imported to establish gene set networks using an overlap coefficient similarity metric (cutoff = 0.5). The AutoAnnotate plugin clustered enriched gene sets into functional modules based on topological relationships and functional similarity, with modules annotated for core biological functions.

#### Relationship between gene expression and stromal proportion

Gene expression values were represented as log_2_-transformed fragments per kilobase of transcript per million mapped reads (FPKM) to approximate a normal distribution. For each target gene, expression levels were compared between the high- and low-fibrosis groups using the Student’s *t* test. Previous study showed that the type I collagen could restrain PDAC growth via mechanical and immune modulation [46]. The tumor-derived collagen IV impedes drug penetration while enhancing proliferation [47]. We analyzed the expression of collagen-related genes (COL1A1, COL1A2, COL4A1, COL4A2, COL4A3, COL4A4, COL4A5, COL4A6) [58,59] in low- and high-fibrosis groups. Moreover, previous studies showed that hENT1(SLC29A1), TUBB3, and LAMC2 were associated with response to gemcitabine [7–10]. We also analyzed the expression of 3 drug sensitivity-related genes (SLC29A1, TUBB3, and LAMC2) in low- and high-fibrosis groups. Additionally, the expression of 3 drug sensitivity-related genes and stromal proportion (as a continuous variable) were analyzed for correlation. Pearson’s correlation was used to calculate the correlation coefficient (*r*) and its corresponding *P* value.

#### Assessment of immune cell infiltration

To evaluate immune cell infiltration levels in PDAC specimens, the MCP-counter method was implemented [60]. Initial normalization involved transforming FPKM values into transcripts per million (TPM) values. Deconvolution was carried out using the deconvo_tme function available in the IOBR R package [61]. Infiltration disparities across cell subsets between low- and high-fibrosis groups were examined using the Student’s *t* test.

### CT-based fibrosis prediction

#### Radiomic feature extraction and selection

Details regarding CT image acquisition, tumor segmentation, and image preprocessing are provided in Supplementary Methods.

Radiomics analysis adhered to the Image Biomarker Standardization Initiative (IBSI) guidelines [62,63], with the overall workflow illustrated in Fig. 5A. Using an open-source PyRadiomics toolkit (version 3.1), we extracted a total of 1,145 radiomic features from the 3D volume of interests encompassing pancreatic tumors in venous-phase CT images, including shape, texture, first-order, and multiscale features. The extracted features were normalized using min–max scaling to ensure comparability across features.

In the SYSUCC training cohort, a 2-step feature selection strategy was used to identify the optimal subset of radiomic features. First, features significantly differing between the low- and high-fibrosis groups were identified using the Student’s *t* test (*P* < 0.05). Next, the least absolute shrinkage and selection operator logistic regression model was applied to select features strongly associated with tumor fibrosis (Table S8).

#### Development and validation of CT-based RM

A binary RM was built using a support vector machine (SVM) classifier to distinguish between high- and low-fibrosis groups based on the selected features. SHAP values were used to interpret the contribution of individual features to model predictions (Fig. 5B).

Model training employed 5-fold cross-validation within the SYSUCC cohort to mitigate overfitting and optimize hyperparameters via grid search. The optimal hyperparameters were determined as penalty coefficient *C* = 1 and radial basis function kernel bandwidth γ = 0.01. The trained model generated continuous probability scores ranging from 0 to 1, with scores ≥0.5 defining high fibrosis. Performance metrics were computed on test folds and aggregated across folds. Among the 5 cross-validated models, the top-performing one was selected for external validation on the independent XYCSU cohort. All analyses were conducted in Python (v3.7) with Scikit-learn (v1.0.2).

#### Association between CT-predicted fibrosis and survival in resectable PDAC

In the SYSUCC and XYCSU surgical cohorts, patients with OS ≥3 months were stratified into high- or low-fibrosis groups using the optimal prediction model selected from 5-fold cross-validation. Kaplan–Meier survival curves and log-rank tests were used to assess the association between CT-predicted fibrosis stratification and prognosis in resectable PDAC.

#### Comparison of different models and CT imaging phases

Using CT imaging data from SYSUCC cohort, this study implemented 4 computational frameworks for fibrosis prediction: (a) a 3D convolutional neural network based on the ResNet architecture, (b) an attention-based multiple instance learning approach using 2.5D slice aggregation, (c) a 2D ResNet-based slice integration model, and (d) radiomic pipelines incorporating conventional machine learning methods (logistic regression, random forest, and XGBoost), as detailed in Supplementary Methods. The performance of these models in predicting fibrosis was systematically compared.

To evaluate the influence of imaging phase on model performance, we constructed SVM classifiers using radiomic features extracted from individual phases (arterial, venous, delayed) as well as from multi-phase images obtained by feature averaging.

### Association between CT-predicted fibrosis and survival across chemotherapy regimens

The CT-based RM was applied to the SYSUCC chemotherapy cohort (*n* = 295) comprising patients receiving AG, FOLFIRINOX, or SOXIRI regimens. Patients were stratified into high- and low-fibrosis groups using the optimal model identified from 5-fold cross-validation. Within each chemotherapy subgroup, differences in OS and PFS between the 2 groups were compared to assess the clinical relevance of CT-predicted fibrosis stratification in patients with PDAC receiving chemotherapy.

Study endpoints for the chemotherapy cohort were OS and PFS. Patients were followed up for survival duration until November 2024. OS was defined as the interval from chemotherapy initiation to death from any cause or last follow-up. PFS was defined as the time from chemotherapy initiation to documented tumor progression (RECIST v1.1), initiation of alternative therapies (surgical resection, irreversible electroporation ablation, or radiation therapy), or all-cause death, whichever occurred first.

### Statistical analysis

Intergroup differences in clinicopathological characteristics were assessed by χ^2^ test, Fisher exact test, or Kruskal–Wallis test, as appropriate. Comparisons of continuous variables between low- and high-fibrosis groups were performed using Student’s *t* test. The performance of the CT-based RM in fibrosis stratification was evaluated by receiver operating characteristic curve and AUC value. Survival analyses were conducted using the Kaplan–Meier method to estimate survival functions, with between-group differences assessed by the log-rank test. All statistical analyses were performed in R (v 4.4.1) using RStudio (v2022.7.1.554) or Python (v3.9.16). Core survival analyses were conducted using the survival package (v3.6.4), with the survfit function for survival curve estimation and the coxph function for Cox modeling. Kaplan–Meier curves were visualized using the ggsurvplot function in the survminer package (v0.4.9), including survival probability plots, risk tables, and annotations of median survival. Immune infiltration results were visualized using ggplot2 (v3.4.4) and ggpubr (v0.6.0) packages. Correlation scatterplots were generated using Python with matplotlib (v3.7.1), seaborn (v0.12.2), and scipy (v1.10.1) packages for data visualization and statistical computation. Statistical significance was defined as *P* < 0.05.

## Ethical Approval

Ethical approval was obtained from the Institutional Review Board of Sun Yat-sen University Cancer Center (approval number: B2022-748-Y02) and Xiangya Hospital of Central South University (transcriptome approval number: 2025081285; CT and pathology data approval number: 2025081314).

## Data Availability

Requests for further information and resources should be directed to and will be fulfilled by the lead contact, J.C. (chengjun583@qq.com). RNA sequencing data are available at https://ngdc.cncb.ac.cn/search/specific?db=bioproject&q=PRJCA040596. For other data used in this research, please email all requests for academic use of raw and processed data to the lead contact (chengjun583@qq.com). All requests will be evaluated based on institutional and departmental policies to determine whether the data requested are subject to intellectual property or patient privacy obligations. Data can only be shared for noncommercial academic purposes and will require a formal materials transfer agreement. All original code has been deposited at https://github.com/HYloong85/PAAD_fibrosis. Any additional information required to reanalyze the data reported in this paper is available from the lead contact upon request.
