# Supplementary material for: Noninvasive Computed Tomography-Based Quantification of Tumor Fibrosis Predicts Pancreatic Cancer Response to Gemcitabine/Nab-Paclitaxel
Source: Research (Wash D C). 2025 Oct 3;8:0937. doi: 10.34133/research.0937 (PMC12491862; doi:10.34133/research.0937)
Supplement: Supplementary 1 — Supplementary Methods Figs. S1 to S4 Tables S1 to S8 [file research.0937.f1.zip › Supplementary Figure 3-new.pdf]

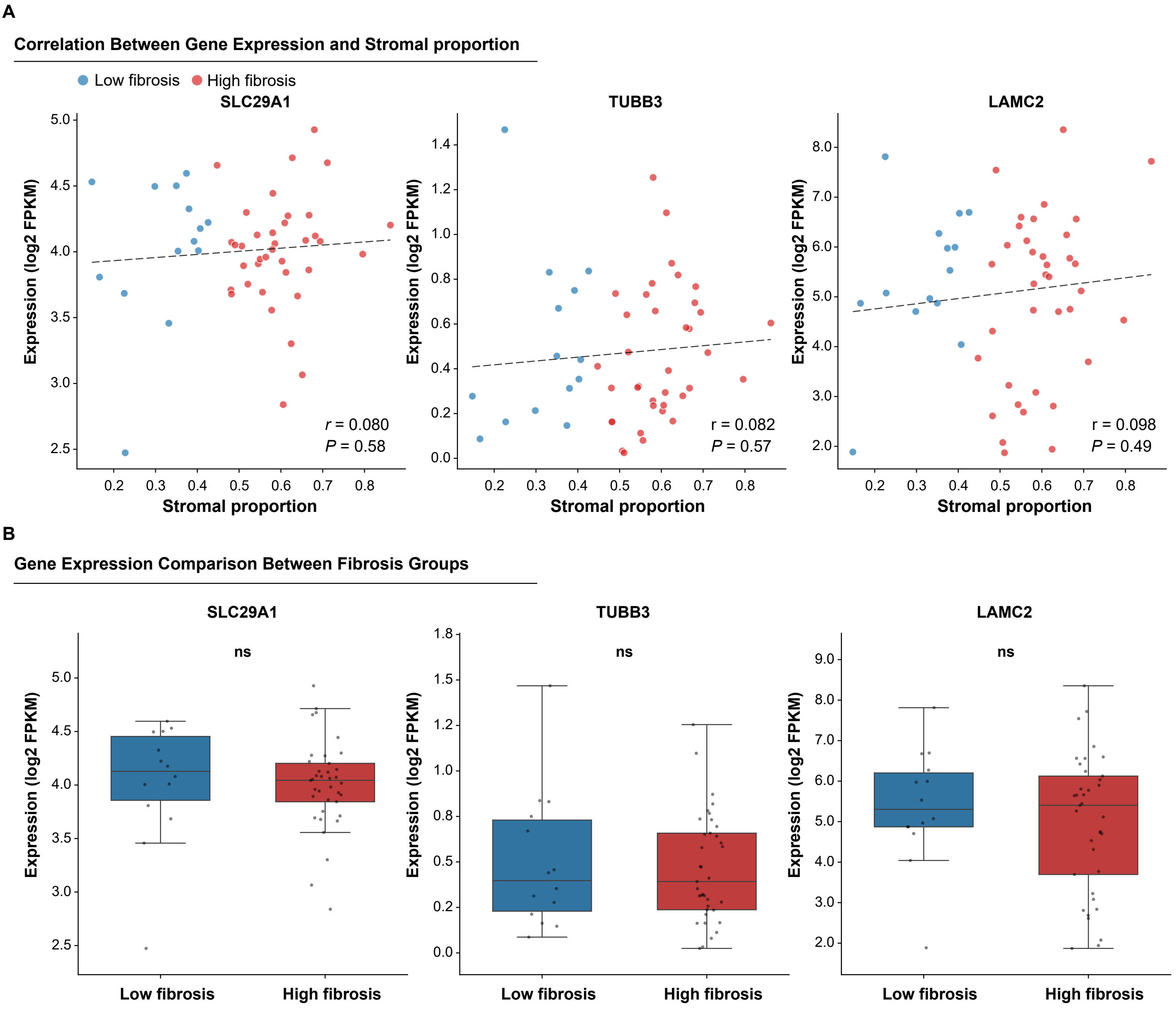
A
Correlation Between Gene Expression and Stromal proportion
Low fibrosis
High fibrosis
SLC29A1
TUBB3
LAMC2
Expression (log2 FPKM)
Stromal proportion
r = 0.080
P = 0.58
r = 0.082
P = 0.57
r = 0.098
P = 0.49
B
Gene Expression Comparison Between Fibrosis Groups
SLC29A1
TUBB3
LAMC2
ns
Expression (log2 FPKM)
Low fibrosis
High fibrosis
