# Supplementary material for: Noninvasive Computed Tomography-Based Quantification of Tumor Fibrosis Predicts Pancreatic Cancer Response to Gemcitabine/Nab-Paclitaxel
Source: Research (Wash D C). 2025 Oct 3;8:0937. doi: 10.34133/research.0937 (PMC12491862; doi:10.34133/research.0937)
Supplement: Supplementary 1 — Supplementary Methods Figs. S1 to S4 Tables S1 to S8 [file research.0937.f1.zip › Supplementary Methods.docx]

**Title: Noninvasive CT-based Quantification of Tumor Fibrosis Predicts Pancreatic Cancer Response to Gemcitabine/Nab-Paclitaxel**

# Supplementary Methods

## Patient enrollment for each study cohort

Between June 2009 and August 2023, 205 patients with pancreatic ductal adenocarcinoma (PDAC) who underwent surgical resection and had available hematoxylin & eosin (H&E)-stained slides were initially identified at SYSUCC. Of these, 35 patients were excluded for tissue artifacts (e.g., folds, tears) or absence of tumor tissue, 17 were excluded for missing preoperative CT imaging or with poor image quality, and 2 were excluded for being lost to follow-up. The final cohort included 151 patients who underwent both contrast-enhanced CT and surgical resection.

Between January 2021 and April 2024, 118 patients with surgically resected PDAC and available H&E-stained slides were identified at XYCSU. The same exclusion criteria as in the SYSUCC cohort were applied, resulting in the exclusion of 28 patients due to loss to follow-up and 3 due to missing preoperative CT imaging. Finally, a total of 87 eligible patients who underwent contrast-enhanced CT and surgical resection were included.

From 2001 to 2013, 185 cases of surgically resected pancreatic adenocarcinoma were retrieved from TCGA. Following exclusion criteria were applied: 2 cases for lack of pathological data; 16 cases not confirmed as PDAC; 43 cases with tissue artifacts (e.g., folds, tears) or absence of tumor tissue; and 1 case lost to follow-up, yielding a final TCGA cohort of 123 patients who underwent surgical resection.

Additionally, 785 patients with unresectable PDAC who received first-line chemotherapy at SYSUCC between October 2013 and June 2023 were initially reviewed. Exclusions included: (1) 223 patients with a history of or concurrent other malignancies, prior antitumor therapy at external institutions, or non-protocol chemotherapy regimens/concurrent immunotherapy at our center; and (2) 267 patients treated with AG, FOLFIRINOX, or SOXIRI regimens but excluded due to incomplete first-line chemotherapy cycles (＜ 3 cycles), baseline pancreatic tumors <10 mm, absence of baseline contrast-enhanced CT imaging (non-contrast CT, MRI, or PET-CT performed instead), lack of follow-up imaging within 3 months after chemotherapy initiation, or incomplete clinical records/loss to follow-up. The final SYSUCC chemotherapy cohort comprised 295 unresectable patients who underwent baseline contrast-enhanced CT and received chemotherapy.

## Treatment and follow-up

For SYSUCC chemotherapy cohort (n=295), all patients received standard chemotherapy following diagnosis. First-line regimens included AG, FOLFIRINOX, or SOXIRI. Subsequent management comprised second-line chemotherapy, observation/supportive care, or surgical resection, irreversible electroporation ablation, radiation therapy. Follow-up CT scans were performed at 2–3 months intervals until death or loss to follow-up.

## Whole-slide image acquisition

Postsurgical H&E-stained specimens were digitized into whole-slide images (WSIs) across three cohorts. WSIs from the TCGA cohort were scanned at ×40 magnification (0.25 μm/pixel resolution) using an Aperio ScanScope scanner. WSIs from the SYSUCC and XYCSU cohorts were scanned at ×20 magnification (0.18 μm/pixel) using a TEKSSQRAY SQS-1000 scanner. All images were stored in SVS format.

All WSIs were manually reviewed. A board-certified pancreatic pathologist (Pathologist 1, with >8 years of experience in pancreatic pathology) confirmed the diagnosis of pancreatic ductal adenocarcinoma and excluded slides exhibiting tissue artifacts (e.g., folds, tears) or lacking tumor tissue. An independent pathologist (Pathologist 2, also with >8 years of experience in pancreatic pathology) verified the WSIs. Any discrepancies were resolved through consensus. Ultimately, one qualified WSI per patient was selected for each cohort (TCGA, SYSUCC, and XYCSU).

## Computed tomography image acquisition

For patients in the SYSUCC cohort, CT examinations were performed using a 64-detector spiral CT system (Discovery CT750 HD, GE System, Milwaukee, WI, USA), a 192-detector spiral CT system (SOMATOM Force CT, Siemens Healthcare, Erlangen, Germany), a 128-detector spiral CT system (Spectral CT 7500, Philips System, Cleveland, OH, USA), or a 80-detector spiral CT system (uCT 780, United Imaging Healthcare, Shanghai, China). The acquisition parameters were as follows: 120 kVp, 150‒300 mA of automatic adjustment, slice thickness of 5 mm, and pitch of 0.984:1. Contrast-enhanced CT images were obtained after a bolus intravenous injection of 1.5 ml/kg nonionic contrast agent (Ultravist 370; Bayer Healthcare, Guangzhou, China; Ioversol 350, Jiangsu Hengrui Pharmaceuticals Co., Ltd., Jiangsu, China; or Iomeprol 350, Bracco Imaging China, Shanghai, China) through an antecubital vein, at a rate of 2.5‒3 ml/s. Multiphase images were obtained at 30‒35 s (arterial phase), 55‒60 s (venous phase), and 180 s (delayed phase). The CT examinations were reconstructed using Standard (soft) kernel.

In the XYCSU cohort, CT examinations were performed using a 64-detector dual-source CT system (SOMATOM Drive, Siemens Healthcare, Erlangen, Germany), a 256-detector spiral CT system (Revolution Apex Expert, GE System, Milwaukee, WI, USA), or a 128-detector spiral CT system (Spectral CT 7500, Philips System, Cleveland, OH, USA). The acquisition parameters were as follows: 120 kVp, 150‒300 mA of automatic adjustment, slice thickness of 5 mm, and pitch of 1.399:1‒0.984:1. Contrast-enhanced CT images were obtained after a bolus intravenous injection of 1.5 ml/kg nonionic contrast agent (Ioversol, Jiangsu Hengrui Pharmaceuticals Co., Ltd., Jiangsu, China; Iohexol, GE Healthcare; Iomeprol, Bracco Imaging China, Shanghai, China) through an antecubital vein, at a rate of 2.5‒3 ml/s. Multiphase images were obtained at 30‒35 s (arterial phase), 55‒60 s (venous phase), and 180 s (delayed phase). The CT examinations were reconstructed using Standard (soft) kernel.

## Volume-of-interest segmentation

The three-dimensional volume of interest (VOI) of the pancreatic tumor was manually delineated by a radiologist with 10 years of subspecialty experience in pancreatic oncology, utilizing the open-source software ITK-SNAP (version 3.8.0, [www.itksnap.org](http://www.itksnap.org/)). VOIs were annotated along tumor margins on each transverse section (slice thickness 5 mm) until complete tumor coverage was achieved. Peripheral vessels or fused lymph nodes were avoided, and necrosis within the tumor was included. To evaluate interobserver reliability, a randomly selected subset of 50 patients from the training cohort underwent independent image segmentation by a second radiologist with 5 years of dedicated pancreatic imaging expertise. Features derived from VOIs delineated by two radiologists demonstrated excellent interobserver agreement (mean interclass correlation coefficient >0.90).

## CT image preprocessing

To mitigate imaging heterogeneity and enhance model robustness, a standardized preprocessing pipeline was implemented: (1) Window normalization: To addressing dynamic range variations in Hounsfield units (HU), a uniform window width of 200 HU and window level of 50 HU were applied. This parameterization optimizes soft-tissue contrast, enhancing density differentiability between pancreatic parenchyma and tumor regions. (2) Spatial resampling: To harmonize resolution differences across scanners, anisotropic resampling standardized voxel dimensions to 0.7mm (x-y plane resolution) × 5mm (inter-slice spacing) using trilinear interpolation. This preserves critical anatomical details while ensuring spatial alignment across multiphase sequences. The preprocessing code was implemented via Python 3.10 (https://www.python.org/) using SimpleITK 2.3.0 (https://simpleitk.org/).

This dual normalization strategy effectively reduces multicenter data heterogeneity, establishing a standardized foundation for radiomic feature extraction and deep learning model training.

## Modeling approaches for fibrosis prediction

This study evaluated the fibrosis prediction performance of four distinct modeling strategies based on CT imaging data: (1) a 3D convolutional neural network (CNN) based on 3D ResNet; (2) a 2.5D network based on attention-based multiple instance learning (AB-MIL); (3) a 2D slice-aggregation learning model based on 2D ResNet; and (4) a radiomics based approach combined with traditional machine learning methods, including logistic regression, random forest, XGBoost, and support vector machine. All experiments were conducted on the SYSUCC surgical cohort using a five-fold cross-validation strategy. Identical dataset splits and preprocessing pipelines were applied across all models to ensuring a fair comparison.

Following preprocessing, the 3D CNN models used the tumor centroid as the cropping center. A region of interest (ROI) measuring 120 × 120 × 16 pixels was extracted based on the largest tumor dimensions (104, 117, 14) in the dataset, then linearly resampled to 224 × 224 × 16 pixels as model input. For 2D deep learning models, tumor-containing slices were extracted, and 120×120-pixel ROIs were centered on the geometric center of each tumor region. Each input slice was constructed as a three-channel image by concatenating the original slice, the original slice multiplied element-wise by its segmentation mask, and the binary mask itself. These inputs were then resized to 224 × 224 × 3 pixels. Identical processing was applied to both the original CT images and corresponding masks. Data augmentation strategies, including random flipping, affine transformations, and random cropping, were applied across all deep learning models to alleviate data scarcity.

To directly quantify fibrotic burden from volumetric CT data, a 3D ResNet-18 was used as the backbone architecture. This model preserves spatial contextual information through volumetric convolutions, enabling direct mapping from 3D grayscale inputs to fibrosis stratification (low and high).

Subsequently, a 2.5D AB-MIL model, was also implemented. This weakly supervised learning framework aggregates slice-level features into patient-level predictions, utilizing a 2D ResNet-18 backbone to extract features from individual CT slices and an attention mechanism to weight and aggregate these features. Each patient’s CT volume was treated as a bag, and tumor-containing slices served as instances within each bag. The attention module assigned weights to individual instances during feature aggregation.

To enhance generalizability through slice-wise integration, a 2D ensemble learning model was developed. This approach decomposed 3D tumor volume into individual 2D slices, each processed using a 2D ResNet-18 with tri-channel inputs identical to the AB-MIL model. All slices from a patient were assigned the same fibrosis label during training, effectively expanding the dataset size. Patient-level predictions were generated by majority voting on slice-level outputs. Although 2D data augmentation helped reduce overfitting, assigning identical labels to all slices introduced slice-level label noise due to intratumoral heterogeneity.

Given the data-hungry nature of deep learning, this study also explored radiomics-based machine learning models. Using radiomic features extracted from preoperative contrast-enhanced CT images, three classifiers—logistic regression, random forest, and XGBoost were implemented and benchmarked against deep learning models.

# The Detailed Legends of Figure 7

**Figure 7** Serial contrast-enhanced CT assessment of treatment response stratified by fibrotic status (high vs. low) and chemotherapy regimens (AG, FOLFIRINOX, and SOXIRI).

**Patient 1 (AG Low-Fibrosis Group):**A 70-year-old female presented with a pancreatic body/tail mass and metastatic lesions in the liver and lungs. At 2.5 months post-chemotherapy, target lesions (pancreatic mass and liver metastases) and non-target lesions (lung metastases) demonstrated significant regression, meeting criteria for partial response (PR). By 4.3 months, progression was observed in the pancreatic mass, partial liver metastases, and lung metastases, yet the overall response remained classified as PR. The patient's progression-free survival (PFS) and overall survival (OS) were both 6.3 months (death).

**Patient 2 (AG High-Fibrosis Group):**
A 51-year-old female presented with a pancreatic head mass and liver metastases. At 4.0 months post-chemotherapy, both the pancreatic mass and liver metastases demonstrated regression, meeting criteria for stable disease (SD). At 7.4 months, the pancreatic mass showed minimal regression while liver metastases exhibited marginal progression, with the overall response maintained as SD. Progressive disease (PD) was confirmed at 9.0 months due to a new metastatic lesion in liver segment 1, despite concurrent regression of existing liver metastases and stable pancreatic findings. PFS was 9.0 months and OS was 17.9 months.

**Patient 3 (FOLFIRINOX Low-Fibrosis Group):**
A 58-year-old male presented with a pancreatic body/tail mass and liver metastases. At 2.0 months post-chemotherapy, both the pancreatic mass and liver metastases demonstrated regression, meeting criteria for PR. By 3.8 months, continued regression of all lesions sustained PR status. PD was confirmed at 7.5 months due to pancreatic mass progression accompanied by a new left upper quadrant peritoneal nodule and upper abdominal ascites (suspected metastatic), despite stable liver metastases. PFS was 7.5 months (treatment switched to AG regimen due to worsening pain); and OS was 14.4 months (censored at last follow-up).

**Patient 4 (FOLFIRINOX High-Fibrosis Group):**
A 60-year-old male presented with a pancreatic tail mass, multifocal liver metastases, and left supraclavicular lymphadenopathy. At 1.9 months post-chemotherapy, regression of the pancreatic mass, liver metastases, and lymphadenopathy met criteria for PR. Sustained regression at 3.8 months maintained PR status. By 5.5 months, a heterogeneous response was observed: minimal pancreatic progression, near-complete resolution of liver segment 8 (S8) lesions with slight progression in S6, and lymph node enlargement, yet overall response remained PR. PD was confirmed at 6.9 months due to pancreatic enlargement, new S8 liver metastasis with concurrent S6 progression, and significant lymph node growth despite persistent S8 resolution. PFS was 6.9 months; and OS was 12.5 months.

**Patient 5 (SOXIRI Low-Fibrosis Group):**A 55-year-old male presented with a pancreatic body mass encasing the celiac trunk, splenic artery, and common hepatic artery, along with bilateral pulmonary metastases. At 2.2 months post-chemotherapy, regression of the pancreatic mass and partial pulmonary metastases met criteria for SD. Persistent SD was maintained at 4.4 months with overall lesion stability. PD was confirmed at 6.5 months due to pancreatic enlargement, progression of pulmonary metastases, and new liver metastases. PFS was 6.5 months; and OS was 11.5 months.

**Patient 6 (SOXIRI High-Fibrosis Group):**A 55-year-old female presented with a pancreatic body mass, multifocal liver metastases, and peritoneal metastases. At 2.7 months post-chemotherapy, regression of the pancreatic mass, liver metastases, and peritoneal nodules met criteria for PR. At 4.7 months, minimal pancreatic and hepatic regression with slight peritoneal progression sustained PR status. PD was confirmed at 6.0 months due to significant progression of liver metastases (including new S2 lesions), peritoneal nodule enlargement, and stable pancreatic findings despite hepatic deterioration. PFS was 6.0 months; and OS was 8.5 months.
