# Supplementary material for: Noninvasive Computed Tomography-Based Quantification of Tumor Fibrosis Predicts Pancreatic Cancer Response to Gemcitabine/Nab-Paclitaxel
Source: Research (Wash D C). 2025 Oct 3;8:0937. doi: 10.34133/research.0937 (PMC12491862; doi:10.34133/research.0937)
Supplement: Supplementary 1 — Supplementary Methods Figs. S1 to S4 Tables S1 to S8 [file research.0937.f1.zip › Supplementary Tables 1-3.docx]

**Supplementary Table 1.** Clinical-pathological characteristics of the patients with resectable PDAC

|  | **TCGA** | **SYSUCC** | **XHCSU** | **P value** |
| --- | --- | --- | --- | --- |
|  | (n = 123) | (n = 151) | (n = 87) |  |
| **Age** (years) |  |  |  | < 0.001 |
| Median | 66 | 60 | 61 |  |
| Range | 35-85 | 37-84 | 42-80 |  |
| **Sex**, n (%) |  |  |  | 0.678 |
| Female | 56 (45.5%) | 62 (41.0%) | 40 (46.0%) |  |
| Male | 67 (54.5%) | 89 (59.0%) | 47 (54.0%) |  |
| **Tumor location**, n (%) |  |  |  | 0.043 |
| Head and/or neck | 98 (79.7%) | 111 (73.5%) | 69 (79.3%) |  |
| Body and/or tail | 25 (20.3%) | 40 (26.5%) | 18 (20.7%) |  |
| **Tumor size** (cm) |  |  |  | 0.268 |
| Median | 3.5 | 3.5 | 3.4 |  |
| Range | 1.5-12 | 0.8-10 | 1-9 |  |
| **AJCC Stage**, n (%) |  |  |  | < 0.001 |
| Stage 1 | 8 (6.5%) | 44 (29.1%) | 42 (48.3%) |  |
| Stage 2 | 109 (88.6%) | 86 (57.0%) | 37 (42.5%) |  |
| Stage 3 | 3 (2.4%) | 17 (11.3%) | 6 (6.9%) |  |
| Stage 4 | 3 (2.4%) | 4 (2.6%) | 2 (2.3%) |  |
| **Differentiation**, n (%) |  |  |  | < 0.001 |
| Well defferentiated | 15 (12.2%) | 0 | 6 (6.9%) |  |
| Moderate defferentiated | 72 (58.5%) | 73 (48.3%) | 60 (69.0%) |  |
| Poorly defferentiated | 36 (29.3%) | 78 (51.7%) | 21 (24.1%) |  |
| **pT stage**, n (%) |  |  |  | < 0.001 |
| T1 | 2 (1.6%) | 15 (9.9%) | 10 (11.5%) |  |
| T2 | 8 (6.5%) | 78 (51.7%) | 46 (52.9%) |  |
| T3 | 111 (90.2%) | 53 (35.1%) | 28 (32.2%) |  |
| T4 | 2 (1.6%) | 5 (3.3%) | 3 (3.4%) |  |
| **pN stage**, n (%) |  |  |  | < 0.001 |
| N0 | 28(22.8%) | 75 (49.7%) | 52 (59.8%) |  |
| N1 | 95 (77.2%) | 65 (43.0%) | 32 (36.8%) |  |
| N2 | 0 | 11 (7.3%) | 3 (3.4%) |  |
| **pM stage**, n (%) |  |  |  | < 0.001 |
| M0 | 66 (53.6%) | 147 (97.4%) | 84 (96.6%) |  |
| M1 | 3 (2.4%) | 4 (2.6%) | 2 (2.3%) |  |
| MX | 54 (44.0%) | 0 | 1 (1.1%) |  |
| **Stroma proportion**, n (%) |  |  |  | < 0.001 |
| High | 52 (42.3%) | 80 (53.0%) | 58 (66.7%) |  |
| Low | 71 (57.7%) | 71 (47.0%) | 29 (33.3%) |  |

PDAC, pancreatic ductal adenocarcinoma; AJCC, American Joint Committee on Cancer.

**Supplementary Table 2.** Clinical characteristics of the patients with unresectable PDAC

|  | **SYSUCC** |  | **AG** | **FOLFIRINOX** | **SOXIRI** | **P value** |
| --- | --- | --- | --- | --- | --- | --- |
|  | (n = 295) |  | (n = 135) | (n = 98) | (n=62) |  |
| **Age** (years) |  |  |  |  |  | 0.039 |
| Median | 58 |  | 60 | 57 | 57 |  |
| Range | 21-78 |  | 30-78 | 21-77 | 34-72 |  |
| **Sex**, n (%) |  |  |  |  |  | 0.107 |
| Female | 118 (40.0%) |  | 61 (45.2%) | 31 (31.6%) | 26 (46.0%) |  |
| Male | 177 (60.0%) |  | 74 (54.8%) | 67 (68.4%) | 36 (54.0%) |  |
| **BMI** |  |  |  |  |  | 0.680 |
| Median | 21.9 |  | 21.8 | 22.1 | 21.6 |  |
| Range | 14.2-37.3 |  | 15.8-30.1 | 15.8-30.3 | 14.2-37.3 |  |
| **ECOG.PS**, n (%) |  |  |  |  |  | 0.548 |
| 0 | 136 (46.1) |  | 62 (45.9%) | 46 (46.9%) | 28 (45.2%) |  |
| 1 | 141 (47.8%) |  | 64 (47.4%) | 49 (50.0%) | 28 (45.2%) |  |
| 2 | 18 (6.1%) |  | 9 (6.67%) | 3 (3.1%) | 6 (9.6%) |  |
| **Tumor location**, n (%) |  |  |  |  |  | 0.025 |
| Head and/or neck | 108 (36.6%) |  | 60 (44.4%) | 32 (32.7%) | 16 (25.8%) |  |
| Body and/or tail | 187 (63.4%) |  | 75 (55.6%) | 66 (67.3%) | 46 (74.2%) |  |
| **Tumor size** (cm) |  |  |  |  |  | 0.824 |
| Median | 42 |  | 43 | 41 | 43 |  |
| Range | 15-123 |  | 21-95 | 15-102 | 21-123 |  |
| **AJCC Stage**, n (%) |  |  |  |  |  | 0.450 |
| Stage 3 | 86 (29.2%) |  | 38 (28.1%) | 26 (26.5%) | 22 (35.5%) |  |
| Stage 4 | 209 (70.8%) |  | 97 (71.9%) | 72 (73.5%) | 40 (64.5%) |  |
| **CA19-9** (U/mL) |  |  |  |  |  |  |
| Median | 547.3 |  | 581 | 882.1 | 224.1 |  |
| Range | 0.6-20000 |  | 1-20000 | 0.6-20000 | 0.6-20000 | 0.147 |
| **Initial chemotherapy response**, n (%) |  |  |  |  |  |  |
| PR | 30 (10.2%) |  | 12 (8.9%) | 11 (11.2%) | 7 (11.3%) |  |
| SD | 215 (72.9%) |  | 107 (79.2%) | 64 (65.3%) | 44 (71.0%) | 0.164 |
| PD | 50 (16.9%) |  | 16 (11.9%) | 23 (23.5%) | 11 (17.7%) |  |
| **Best overall response**, n (%) |  |  |  |  |  |  |
| PR | 84 (28.5%) |  | 31 (22.9%) | 30 (30.6%) | 23 (37.1%) |  |
| SD | 161 (54.6%) |  | 88 (65.2%) | 45 (45.9%) | 28 (45.2%) | 0.164 |
| PD | 50 (16.9%) |  | 16 (11.9%) | 23 (23.5%) | 11 (17.7%) |  |
| **CT- predicted fibrotic status**, n (%) |  |  |  |  |  |  |
| High | 162 (54.9%) |  | 76 (56.3%) | 50 (51.0%) | 36 (58.1%) |  |
| Low | 133 (45.1%) |  | 59 (43.7%) | 48 (49.0%) | 26 (41.9%) | 0.621 |
| **PFS** (months) |  |  |  |  |  |  |
| Median | 5.03 |  | 4.77 | 4.67 | 5.75 |  |
| Range | 1.00-21.57 |  | 1.00-19.97 | 1.30-16.07 | 1-21.57 | 0.097 |
| **OS** (months) |  |  |  |  |  |  |
| Median | 10.97 |  | 10.6 | 11.82 | 11.67 |  |
| Range | 1.33- 69.43 |  | 1.43-69.43 | 1.87-49.40 | 1.33-50.90 | 0.987 |

PDAC, pancreatic ductal adenocarcinoma; AJCC, American Joint Committee on Cancer; BMI, body mass index; ECOG.PS, Eastern Cooperative Oncology Group Performance Status; PR, partial response; SD, stable disease; PD, progressive disease; PFS, progression-free survival; OS, Overall survival.

**Supplementary Table 3.** Numbers of features that remained after each selection step during radiomics model building

| **Feature selection steps** | **Numbers of features remaining** |
| --- | --- |
| Before selection | 1145 |
| Student’s t-test | 86 |
| LASSO regression | 15 |

LASSO: least absolute shrinkage and selection operator.
