# Supplementary material for: Noninvasive Computed Tomography-Based Quantification of Tumor Fibrosis Predicts Pancreatic Cancer Response to Gemcitabine/Nab-Paclitaxel
Source: Research (Wash D C). 2025 Oct 3;8:0937. doi: 10.34133/research.0937 (PMC12491862; doi:10.34133/research.0937)
Supplement: Supplementary 1 — Supplementary Methods Figs. S1 to S4 Tables S1 to S8 [file research.0937.f1.zip › Supplementary Tables 6-8.docx]

**Supplementary Table 6.** The 15 radiomic features and LASSO coefficients for the radiomics model

| **Radiomic Features** | **Coefficients** |
| --- | --- |
| original_shape_Compactness1 | 0.002172 |
| original_shape_Compactness2 | 0.021529 |
| log-sigma-2-mm-3D_firstorder_Kurtosis | 0.054305 |
| log-sigma-2-mm-3D_glcm_Imc1 | 0.028207 |
| log-sigma-3-mm-3D_firstorder_Skewness | 0.005495 |
| log-sigma-3-mm-3D_glszm_GrayLevelVariance | 0.034190 |
| log-sigma-3-mm-3D_glszm_LargeAreaLowGrayLevelEmphasis | -0.036084 |
| log-sigma-5-mm-3D_glszm_SmallAreaEmphasis | -0.079476 |
| wavelet-LLH_firstorder_Skewness | 0.079918 |
| wavelet-LLH_ngtdm_Busyness | -0.003095 |
| wavelet-LHL_firstorder_Skewness | 0.046747 |
| wavelet-HLL_glcm_Imc2 | 0.005696 |
| wavelet-HLL_glrlm_RunVariance | -0.017262 |
| wavelet-HHH_glcm_Imc1 | 0.056142 |
| wavelet-LLL_glszm_LargeAreaHighGrayLevelEmphasis | -0.007190 |

**Supplementary Table 7.** Predictive performance of five cross-validation models in training and external test cohorts

| **Datasets** | **Methods** | **AUC**  (95%CI) | **Accuracy** (95%CI) | **F1 score** (95%CI) |
| --- | --- | --- | --- | --- |
| **SYSUCC** | **Fold-1** | **0.773**  **(0.653, 0.889)** | **0.767**  **(0.667, 0.867)** | **0.811**  **(0.718, 0.895)** |
|  | Fold-2 | 0.751  (0.629, 0.869) | 0.733  (0.633, 0.833) | 0.733  (0.600, 0.837) |
|  | Fold-3 | 0.685  (0.561, 0.809) | 0.667  (0.567, 0.767) | 0.649  (0.529, 0.756) |
|  | Fold-4 | 0.652  (0.506, 0.787) | 0.600  (0.500, 0.703) | 0.647  (0.500, 0.757) |
|  | Fold-5 | 0.819  (0.710, 0.923) | 0.633  (0.500, 0.733) | 0.605  (0.462, 0.696) |
|  | Average±SD | 0.736 ±0.060 | 0.680 ±0.062 | 0.689 ±0.074 |
| **XYCSU** | **Fold-1** | **0.718**  **(0.644, 0.782)** | **0.678**  **(0.621, 0.736)** | **0.785**  **(0.735, 0.830)** |
|  | Fold-2 | 0.692  (0.616, 0.762) | 0.567  (0.498, 0.636) | 0.782  (0.726, 0.832) |
|  | Fold-3 | 0.710  (0.634, 0.785) | 0.667  (0.609, 0.736) | 0.775  (0.721, 0.825) |
|  | Fold-4 | 0.714  (0.637, 0.789) | 0.644  (0.586, 0.713) | 0.767  (0.716, 0.818) |
|  | Fold-5 | 0.695  (0.617, 0.766) | 0.667  (0.598, 0.724) | 0.800  (0.748, 0.840) |
|  | Average±SD | 0.706 ±0.010 | 0.644 ±0.040 | 0.782 ±0.011 |

AUC, area under the ROC curve; CI, confidence interval; SD, standard deviation.

**Supplementary Table 8.** Comparison of different models and CT phases

|  | **Methods** | **AUC**  (Average±SD) | **Accuracy** (Average±SD) | **F1 score**  (Average±SD) |
| --- | --- | --- | --- | --- |
| **Model comparison** | **SVM** | **0.736±0.060** | **0.680±0.062** | **0.689 ±0.074** |
|  | RF | 0.657±0.110 | 0.648±0.110 | 0.6568±0.0677 |
|  | LR | 0.681±0.056 | 0.723±0.038 | 0.681±0.055 |
|  | XGBoost | 0.707±0.104 | 0.694±0.071 | 0.653±0.075 |
|  | 3DResnet | 0.570±0.010 | 0.585±0.048 | 0.578±0.051 |
|  | ABMIL | 0.645±0.069 | 0.648±0.071 | 0.630±0.083 |
|  | 2DResnet | 0.677±0.076 | 0.681±0.055 | 0.677±0.059 |
| **CT phases** | **Venous** | **0.736±0.060** | **0.680±0.081** | **0.685±0.086** |
|  | Arterial | 0.698±0.068 | 0.647±0.102 | 0.561±0.099 |
|  | Delayed | 0.639±0.063 | 0.689±0.078 | 0.565±0.102 |
|  | Triple phases | 0.709±0.094 | 0.704±0.062 | 0.686±0.070 |

AUC, area under the ROC curve; SVM, RF, random forest; LR, logistic regression; ABMIL, attention-based multiple instance learning.
