# Supplementary figures and images for: Noninvasive Computed Tomography-Based Quantification of Tumor Fibrosis Predicts Pancreatic Cancer Response to Gemcitabine/Nab-Paclitaxel
Source: Research (Wash D C). 2025 Oct 3;8:0937. doi: 10.34133/research.0937 (PMC12491862; doi:10.34133/research.0937)

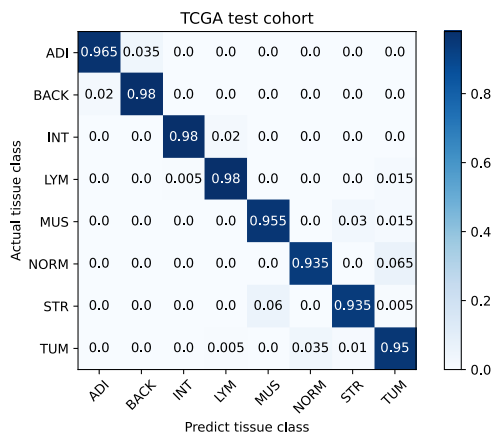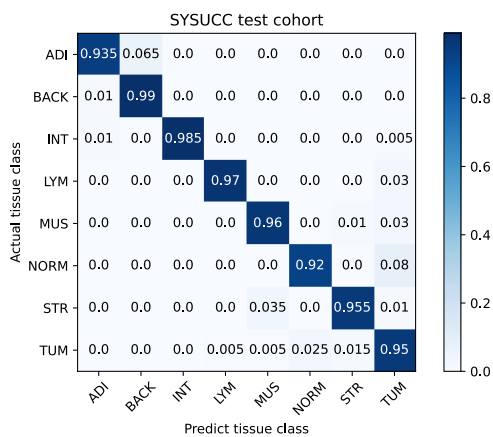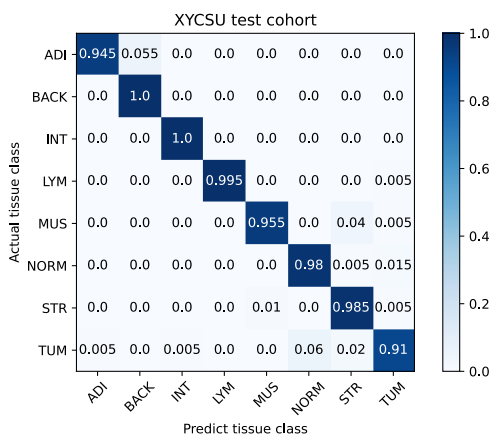

Supplement: Supplementary 1 — Supplementary Methods Figs. S1 to S4 Tables S1 to S8 [file research.0937.f1.zip › Supplementary Figure 1.pdf]

**A**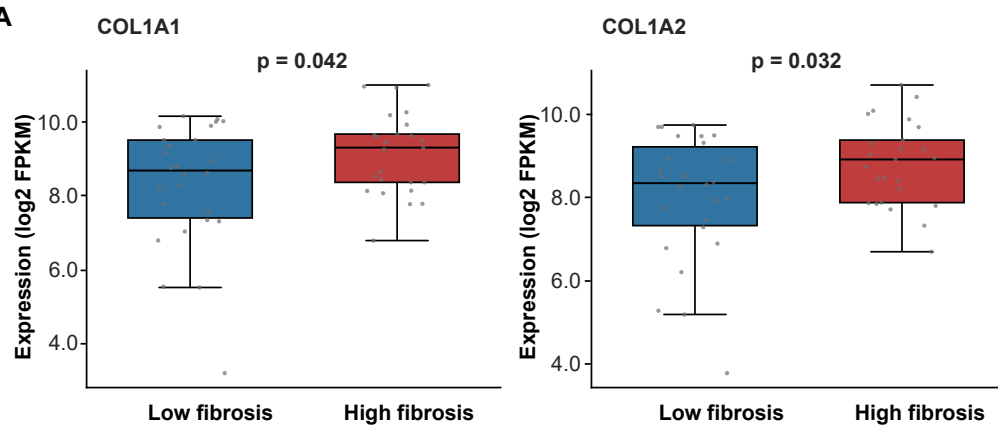**B**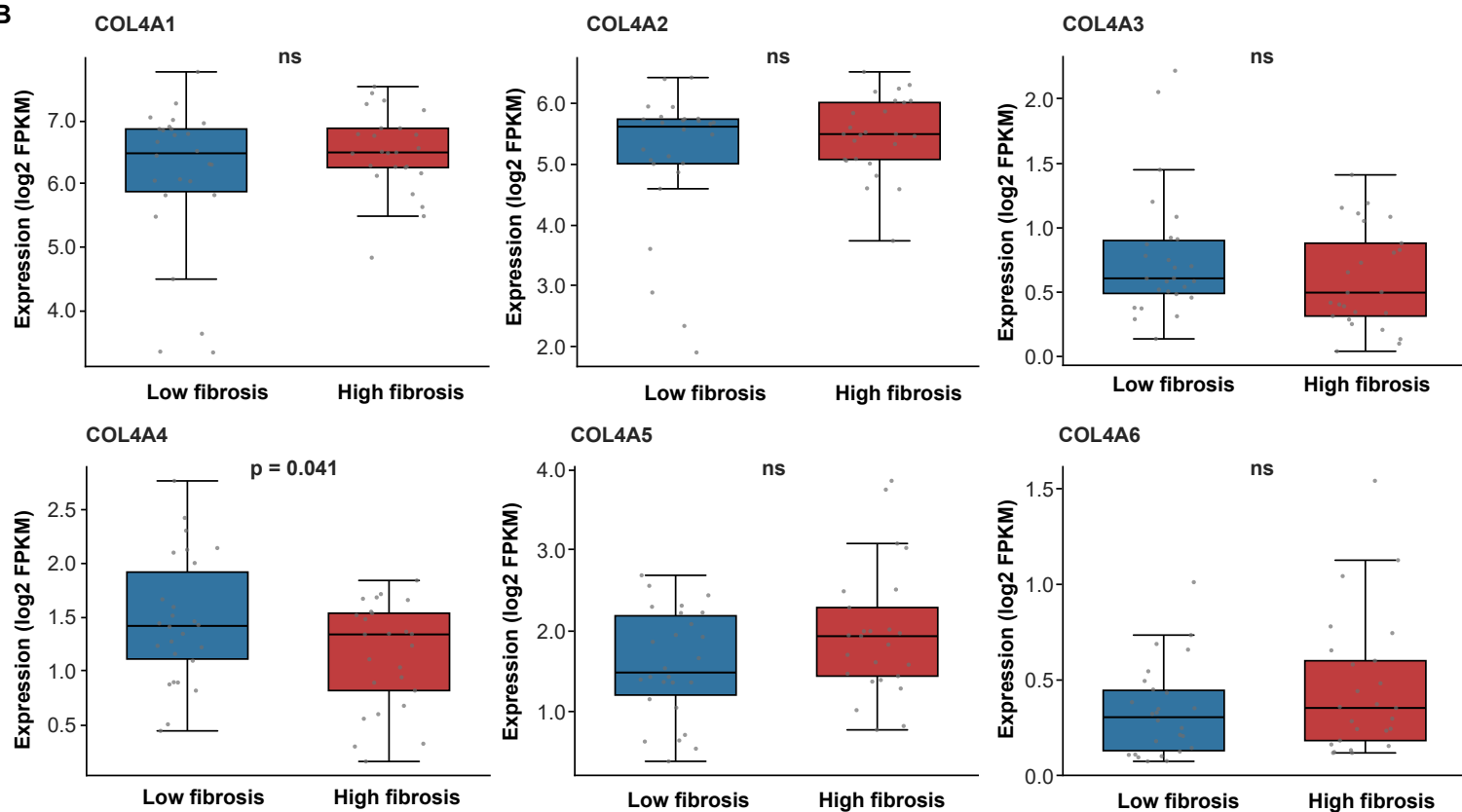**C**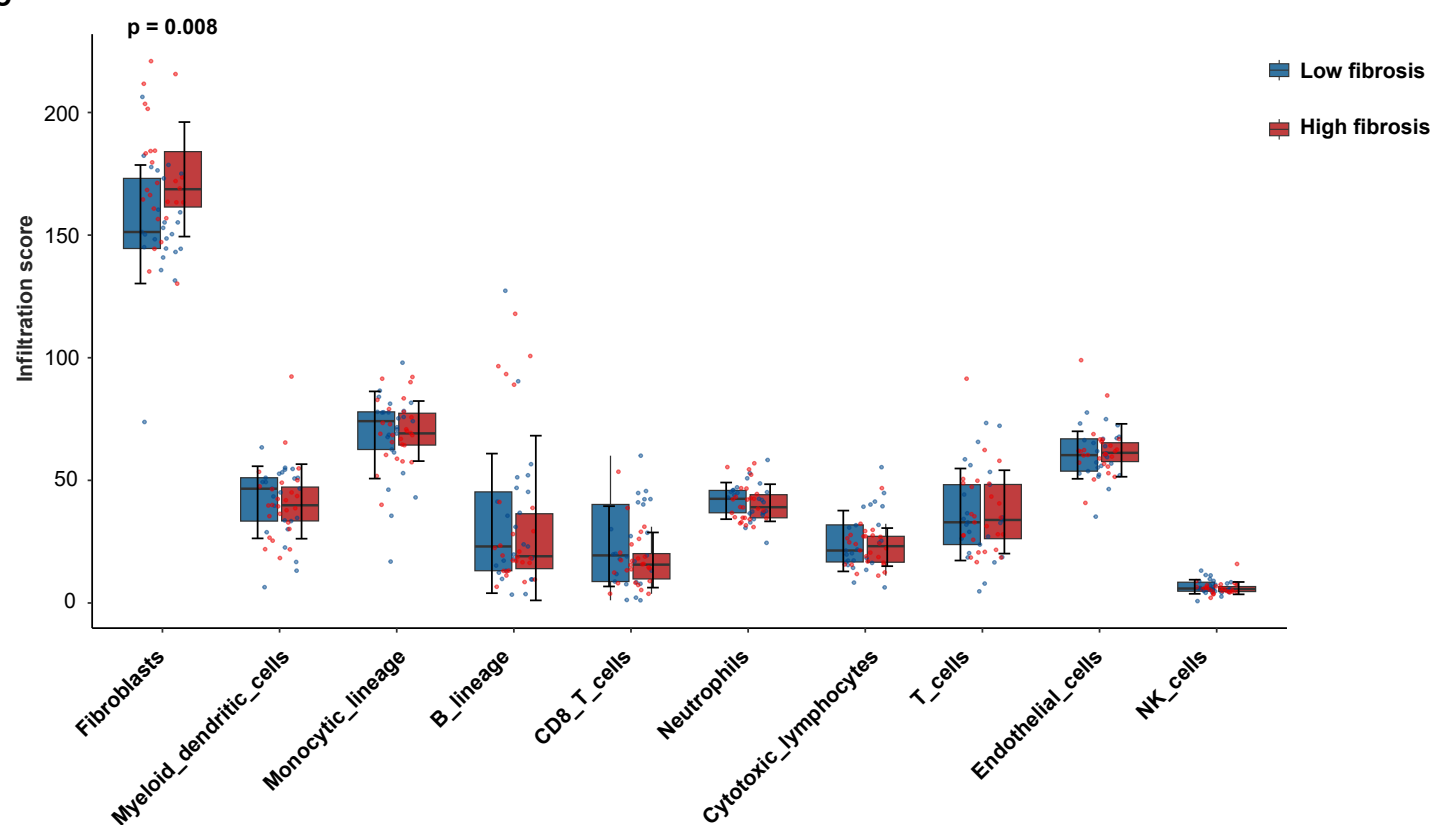

Supplement: Supplementary 1 — Supplementary Methods Figs. S1 to S4 Tables S1 to S8 [file research.0937.f1.zip › Supplementary Figure 2-new.pdf]

SYSUCC

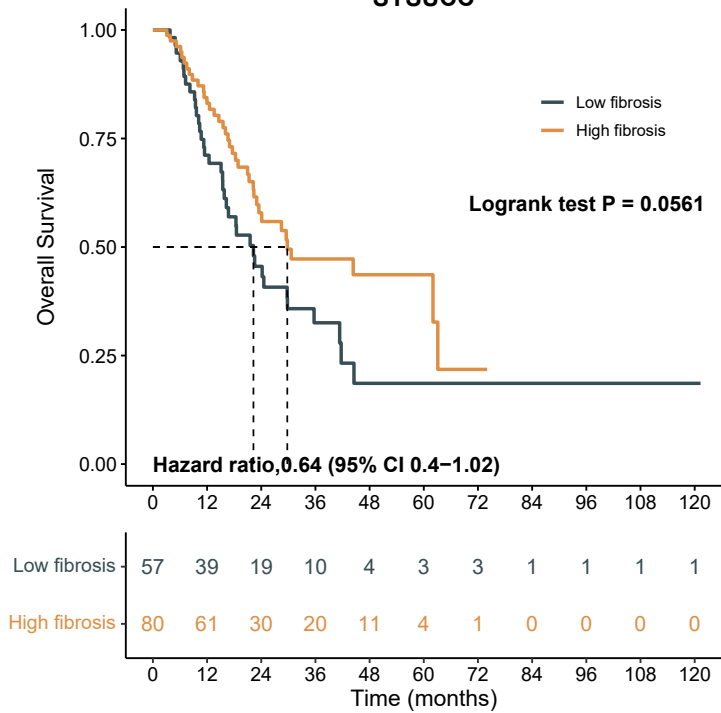

XYCSU

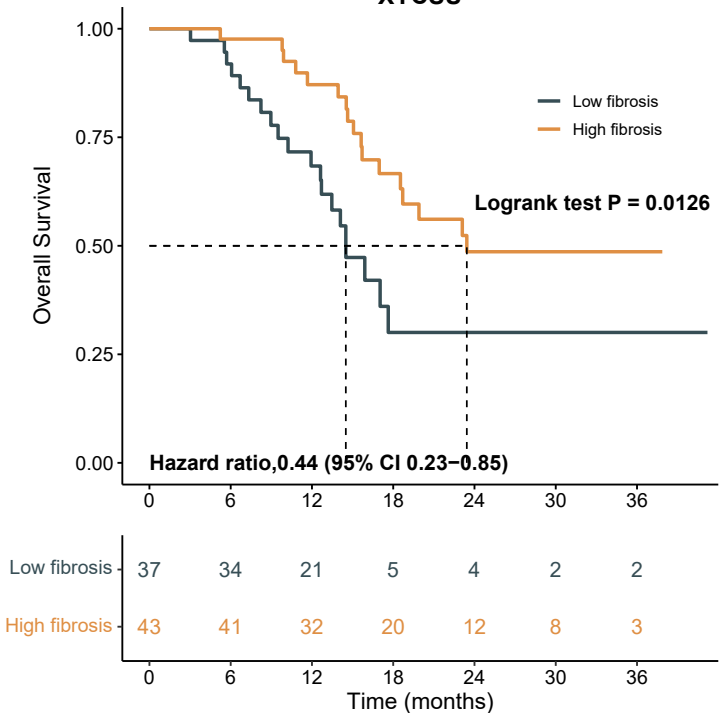

Supplement: Supplementary 1 — Supplementary Methods Figs. S1 to S4 Tables S1 to S8 [file research.0937.f1.zip › Supplementary Figure 4.pdf]
